# Sleep Quality Assessment and Its Predictors Among Saudi Adults with Type 1 and Type 2 Diabetes: A Cross-Sectional Study

**DOI:** 10.3390/ijerph21111437

**Published:** 2024-10-29

**Authors:** Abdulaziz A. Alhoqail, Khaled H. Aburisheh, Abdulrahman M. Alammar, Mohammed A. Bin Mugren, Abdulrahman M. Shadid, Ibrahim K. Aldakhil, Hamza M. K. Enabi, Faisal N. Alotaibi

**Affiliations:** 1 Meena Health Care, Riyadh 13525, Saudi Arabia; aalhoqail@meena-health.com; 2 Consultant of Medicine & Diabetes, University Diabetes Center, King Saud University Medical City, King Saud University, P.O. Box 11472, Riyadh 7805, Saudi Arabia; 3 University Family Medicine Center, Department of Family and Community Medicine, College of Medicine, King Saud University Medical City, P.O. Box 11472, Riyadh 7805, Saudi Arabia; aalammar1@ksu.edu.sa (A.M.A.); mbinmugren@ksu.edu.sa (M.A.B.M.); 4 Internal Medicine Department, Dr. Sulaiman Al Habib Group, Riyadh 13325, Saudi Arabia; abm.shadid@gmail.com; 5 Internal Medicine Department, College of Medicine, King Saud University, P.O. Box 11472, Riyadh 7805, Saudi Arabia; ibrahimkd20@gmail.com; 6 College of Medicine, Alfaisal University, P.O. Box 50927, Riyadh 11533, Saudi Arabia; henabi@alfaisal.edu; 7 Department of Family and Community Medicine, Armed Forces Hospital, P.O. Box 413, Jubail 31951, Saudi Arabia; fnba85@gmail.com

**Keywords:** diabetes mellitus, sleep quality, glycemic control, Pittsburgh Sleep Quality Index

## Abstract

**Background:** Poor sleep quality is prevalent among adults with diabetes, impacting their physical, psychological, and functional well-being. Our goals were to assess the prevalence of poor sleep quality and its association with glycemic control and to identify predictors of poor sleep quality among adults with diabetes mellitus. **Methods:** This cross-sectional study took place at a tertiary hospital, from October 2022 to March 2023, including 192 adults with type 1 and type 2 diabetes. We collected demographic and clinical data and utilized the Pittsburgh Sleep Quality Index (PSQI) scale to evaluate sleep quality. **Results:** A total of 65.9% of the participants experienced poor sleep quality (PSQI score > 5), with an average global sleep quality score of 7.36 ± 3.53 for all patients. Poor sleep quality was higher among older and married patients, those with lower education levels, housewives, and those with type 2 diabetes mellitus and associated comorbidities, such as hypertension and dyslipidemia. None of these factors were significantly associated with sleep quality in a multiple linear regression analysis. The mean glycated hemoglobin was 8.68 ± 1.91% and did not correlate with the overall PSQI score and its components. **Conclusions:** This study revealed a notably high prevalence of poor sleep quality among Saudi adults with diabetes, potentially associated with specific sociodemographic and clinical factors. These findings emphasize the importance of integrating sleep quality education into diabetes management strategies.

## 1. Introduction

Diabetes mellitus (DM) is a crucial public health problem. It is associated with a decrease in life expectancy; the significant morbidity associated with DM arises from microvascular and macrovascular complications, including ischemic heart disease, stroke, peripheral vascular disease, and worsening quality of life [1]. It was estimated that around 10.5% of the global population was affected by DM, according to the International Diabetes Federation report 2021 [2], and the World Health Organization (WHO) has reported that Saudi Arabia has the second-highest rate of DM in the Middle East and seventh-highest in the world [3]. It is estimated that approximately 7 million people in the population have DM, and almost 3 million have pre-diabetes [3]. 

Poor sleep quality and sleep disorders, mainly insomnia, are common problems in primary care [4]. In the Saudi population, 33% of middle-aged Saudi males are at risk for obstructive sleep apnea [5], and 72.5% of Saudi adults experience poor sleep quality [6]. Sleep quality and duration significantly affect the physical and mental quality of life of those within the general population [7]. People with DM were more likely than those without DM to report sleep problems such as sleep apnea and inadequate sleep [8]. Evidence suggests that sleep disorders may be considered a risk for DM, and conversely, DM itself could contribute to sleep disorders [9]. Moreover, the risk of poor sleep quality was found to be greater in adults with type 2 DM (T2DM) compared to those with type 1 DM (T1DM) [10]. The improvement of poor sleep quality in patients with DM is essential, as it has been recognized as one of the causes that can lead to several complications experienced by patients with diabetes. Poor sleep quality is related to poor physical, mental, and functional consequences [11].

Sleep quality could be one of the independent risk factors of uncontrolled DM [12]. However, there are controversial results regarding the association between glycated hemoglobin (HbA1c) and sleep disturbance [13]. Sleep interventions would be non-pharmacological, inexpensive, and applicable in a limited-resource setting [4].

Different available scales can assess sleep quality, including the Pittsburgh Sleep Quality Index (PSQI). This scale was introduced by Buysse et al. and found to be a reliable and validated tool (Cronbach’s alpha of 0.83) [14]. It was tested and validated in different clinical and non-clinical populations [15]. DM is one of the diseases in relation to which this tool has evaluated the impact of sleep quality on glycemic control in many studies [12].

Few studies measure the prevalence of sleep quality in adults with DM in our region. Through this study, we aim to assess the prevalence of poor sleep quality in adults with DM using the PSQI tool. Additionally, we aim to evaluate the correlation between sleep quality and HbA1c and to investigate the factors associated with poor sleep quality among adults with T1DM and T2DM. The findings of this study will provide valuable insights into the relationship between sleep quality and DM, potentially leading to improved patient care and outcomes.

## 2. Materials and Methods

This cross-sectional study was conducted from October 2022 to March 2023 at diabetes clinics in King Saud University Medical City (KSUMC), Riyadh, Saudi Arabia. It was conducted in strict adherence to the relevant guidelines and regulations, and the Institutional Review Board of the College of Medicine, King Saud University, Saudi Arabia, reviewed and approved the protocol (E-22-6979). 

### 2.1. Participants

The study’s targeted population was carefully selected, focusing on adults with T1DM and T2DM who were 18 years old and above and able to communicate in Arabic and visit adult diabetes clinics at the diabetic center. We excluded pregnant patients and those with iron deficiency anemia, hemoglobinopathies, chronic kidney disease, and those who had a recent blood transfusion.

The study’s required sample size was calculated using a rigorous simple random sampling technique with a 95% confidence interval, resulting in a robust sample of 351 patients. However, due to some limitations during sample collection, mainly patient acceptance and compliance with answering the survey, we retained 192 patients in our study.

### 2.2. Procedure

The questionnaire prepared for the study consisted of two parts. The first part was prepared by the study’s authors, and the second part was the PSQI questionnaire. The first part of the questionnaire consisted of the demographic and clinical variables, including age, biological sex, marital status (dichotomized as single, married, divorced, and widowed), educational status (categorized into seven groups: illiterate, ”those who cannot read and write”; R&W, “those who can only read and write”; primary; intermediate; secondary; diploma; and university), type of job, smoking status, exercise pattern (defined as the number of hours spent exercising per week), coffee intake (defined as the number of cups of coffee ingested per day and the time of the last cup ingested in the day), medical history, DM complications (defined as the presence of any micro- and macrovascular complications of DM), insulin usage, hypoglycemia history (defined as the presence of hypoglycemia within the last month), type of DM (dichotomized as T1DM and T2DM), DM duration, and HbA1c. We used the Arabic-translated version of PSQI with an internal consistency reliability of 0.74 [16] after obtaining permission from the authors. We used this tool as it is reliable and validated, having been tested in the DM population in many studies. This scale is a self-administered 19-item instrument that measures various aspects of sleep quality and disturbance over the previous month, including seven subscales: sleep quality, sleep latency, sleep duration, habitual sleep efficiency, sleep disturbances, use of sleeping medication, and daytime dysfunction. Each subscale was scored from 0 to 3, and the sum of all scores gave a PSQI global score ranging from 0 to 21. A greater score indicated poorer sleep quality. A cutoff score of 5 was used in this study to differentiate poor sleepers (>5) from good sleepers (≤5), with a sensitivity and specificity of 89.6 and 86.5, respectively [11]. The informed consent form was explained to the patients and signed before the questionnaire was provided. The data were collected from patients and their electronic medical records, and the questionnaire that patients attending clinics at the University Diabetes Center were to fill out was distributed and explained. The latest HbA1c results were collected from patients’ electronic files.

### 2.3. Statistical Analyses

Analyses were performed using (PSPP version 1.6.2-g78a33a). Data were demonstrated by mean and SD or number and percentage for numerical and categorical variables, respectively. The normality of the data was tested using the Shapiro–Wilk test, and the data were found to be not normally distributed. Demographic and clinical data were compared between poor and good sleepers and between adults with T1DM and T2DM. The Wilcoxon–Mann–Whitney test for continuous variables and Chi-squared test for categorical variables. A non-parametric Spearman correlation was used to examine the correlation between HbA1c and each subscale score (continuous variables). Finally, a multiple linear regression analysis was performed to evaluate the independent association of the variables of interest with the PSQI global score. All *p*-values less than 0.05 were considered statistically significant.

## 3. Results

The total number of patients who answered the survey was 192; however, 182 completed PSQI questionnaires were received, giving a response rate of 94.8%. We found that 34.1% of the participants were good sleepers, and 65.9% were poor sleepers. The demographic and clinical characteristics of the study participants shown in Table 1 and Supplementary Table S1 indicated that the mean study participants’ age was 40.95 ± 20.04 years, with significantly younger ages (35.58 ± 19.29 years) among good sleepers compared to poor sleepers (43.89 ± 19.95 years, *p* = 0.011), and among patients with T1DM (29.82 ± 17.19 years) compared to those with T2DM (55.26 ± 13.22, *p* = 0.000). The distribution according to biological sex was almost equal, with no significant difference according to sleep quality (*p* = 0.754) and the types of DM (*p* = 0.363). Significantly, the majority of the good sleepers were single, while the majority of the poor sleepers were married (*p* = 0.008). Furthermore, single marital status was significantly more common in those with T1DM, and 76.2% of those with T2DM were married (*p* = 0.000).

Moreover, there was a significant difference between the poor and good sleep quality groups in terms of education level, as a lower education level had a greater rate of poor sleep quality. However, intermediate and secondary education levels were found to be higher in good sleepers (*p* = 0.022). Furthermore, one-third of the study population had a job, which was full-time for 76% of them, and being a student was more common among good sleepers; however, being a housewife was more common among poor sleepers (*p* = 0.005). Similarly, the majority of those with T1DM were students, and the majority of those with T2DM were housewives (*p* = 0.000). In contrast, there was no significant association between job type and sleep quality or DM type (*p* = 0.784 and *p* = 0.201, respectively). 

Nearly one-third of the study population had hypertension (HTN), dyslipidemia, and DM complications, with a significantly higher prevalence of HTN and dyslipidemia in the poor sleepers (*p* = 0.005 and *p* = 0.015, respectively) and in those with T2DM (*p* = 0.000 for both comorbidities). However, there was no significant difference in other comorbidities (pulmonary disease, thyroid disease, and psychiatric disease) between the groups. Reported DM complications were significantly higher in adults with T2DM compared to those with T1DM (51.2% vs. 30.6%, *p* = 0.004), while there was no significant difference between the sleep quality groups.

We also found high insulin usage in the study population (81.3%), which was significantly greater in those with T1DM (*p* = 0.000), with no significant difference between good and poor sleepers (*p* = 0.3). Moreover, 112 participants (58.3%) reported hypoglycemia, which was reported significantly more often among the good sleepers (*p* = 0.049) and those with T1DM (*p* = 0.000).

The study participants were distributed almost equally according to the type of DM, with a mean DM duration of 14.06 ± 9.66 years, which was significantly longer in the adults with T2DM (*p* = 0.005); moreover, there was no significant difference between the sleep quality groups (*p* = 0.11). T1DM was observed to be more common among the good sleepers with a lower global PSQI score (6.65 ± 3.09), while T2DM was more common among the poor sleepers with a higher PSQI score (8.28 ± 3.85) (*p* = 0.049 for sleep quality difference, and *p* = 0.005 for PSQI score difference). The mean HbA1c level for the participants in this study was 8.68 ± 1.91%, which was equal in all the groups.

Table 2 represents the distribution of the PSQI subcomponents among the study participants. The average global PSQI score for all of the study population was 7.36 ± 3.53, which indicates poor sleep quality. The highest-scoring of the PSQI subcomponents in this study was sleep latency, with an average score of 1.49 ± 1.01, suggesting that these patients take longer to fall asleep. On the other hand, the lowest score was for the use of sleep medication, with an average score of 0.37 ± 0.83, as 81.1% of the participants did not rely on medications to sleep. Subjective sleep quality had an average score of 1.02 ± 0.77, with 23.5% of the participants reporting very good and 56.1% reporting fairly good sleep quality.

Table 3 shows the correlation between the study participants’ glycemic control and PSQI subcomponent scores. The findings indicated no significant correlations between the level of HbA1C and any of the PSQI subscales. A multiple linear regression analysis was performed to determine the factors that could be independently associated with the PSQI global score, as shown in Table 4. In the analysis, we included the variables that were found to have a significant difference in the sleep quality prevalence in our study. The results showed that marital status, age, education, job status, HTN, dyslipidemia, the presence of hypoglycemia, DM type, and HbA1C had no significant associations with sleep quality.

## 4. Discussion

In this research work, we aimed to elucidate the relationship between sleep quality and various demographic and clinical characteristics in adults diagnosed with T1DM and T2DM. This study uniquely contributes to the body of knowledge by identifying specific sociodemographic and clinical factors that are associated with a higher prevalence of poor sleep quality, such as older age, marital status, lower educational level, and specific comorbid conditions. These findings underscore the complexity of sleep disturbances in populations with DM and highlight the need for a multifactorial approach to patient management strategies. This resonates with studies such as those by Al Ahmari et al., emphasizing the multifaceted nature of sleep disturbances across diabetic complications [17]. For instance, Darraj et al. conducted a systematic review highlighting that sleep disorders and T2DM are prevalent and frequently coexist. The findings of this review suggest that sleep problems can exacerbate metabolic abnormalities, including T2DM, and vice versa, complicating patient management [18]. Additionally, Al Nefaie et al. demonstrated that poor sleep quality is significantly associated with various determinants, such as the duration of diabetes and social stressors. The findings of their study further reinforce the necessity of integrating sleep assessment and management into standard diabetic care practices, emphasizing that these measures are crucial for effective glycemic control and overall patient well-being [19]. 

Using the most commonly recommended measure of sleep quality (PSQI), we found the prevalence of poor sleepers within our study population to be 65.9%, with an average global score of 7.36, which is lower than that of the general Saudi population (around 75%) [6,20]. Sleep latency was the most affected component in the PSQI, followed by sleep duration, as almost one-third of the participants slept more than 7 h daily. Moreover, 81% of them did not rely on sleep medication. Our findings mostly correlate with Rajendran et al. and Jeong et al.’s results [21,22]. In the literature, the prevalence of poor sleep quality in patients with DM ranges from 23% to 97% [17,19,21,22,23,24,25]. This wide range could be explained by the differences in the definition criteria of poor sleep quality and in the demographic characteristics such as age, educational level, marital status, and social stressors between the studies. Moreover, most of the studies included only patients with T2DM; however, we included both types. Finally, the period when the studies were performed could have affected the sleep quality, as there was an observable a significant reduction in sleep quality during the Coronavirus Disease 2019 pandemic [26].

The results of this study indicate that younger patients generally reported better sleep than older ones, which aligns with findings from other studies [27,28,29,30]. These studies collectively indicate that age is a critical determinant of sleep quality in populations with DM. The progressive nature of DM, combined with the physiological and psychological stresses associated with ageing, underscores the importance of incorporating age-specific strategies in DM management to improve sleep health and overall quality of life for these patients.

Intriguingly, our study found that married participants reported poorer sleep quality compared to their single counterparts, possibly reflecting the increased psychosocial stress associated with the responsibility of family life coupled with disease management. Similar findings were observed by Barakat et al. and Zewdu et al. [25,29]. This finding suggests a complex interplay between personal relationships and chronic disease management, where social stressors were significantly linked to poor sleep quality [17,19].

Among our participants, those with higher educational levels reported better sleep quality, supporting many studies [28,29,30] that noted a similar trend among their study populations. This indicates that education equips individuals with better resources and skills for effective disease management and stress-coping mechanisms, which can directly impact sleep quality by moderating anxiety and improving self-care behaviors. In contrast, Edmealem et al. found that higher educational levels were linked to poorer sleep quality among patients with chronic illnesses, including DM, HTN, and heart failure. They explained their results by the increase in various social stressors. However, their results showed that patients who had better perceptions of their illness prognosis were more likely to practice effective stress management strategies, contributing to improved sleep quality [31].

There is controversial evidence regarding the correlation between employment status and sleep quality. However, most of the studies defined job status as employed and unemployed [17,19,25,31]. Our finding is in line with the studies that showed a significant correlation, as we found that housewives were poorer sleepers. Khosravan et al. included housewives as a subgroup of the job variable and obtained a similar finding [32]. This result could be explained by cultural factors, social stressors, and the exhausting domestic responsibilities of women in our region. A study on the Saudi population showed that women who are housewives experienced more sleep disturbance than other job groups [33].

Our study highlights a clear link between sleep quality and comorbid conditions, mainly hypertension and dyslipidemia, emphasizing the additive burden of multiple chronic conditions on overall health, particularly sleep. Similar findings have been documented in other studies [17,29,34]. Agyekum et al. found that poor sleep quality in adults with T2DM was associated with low HDL cholesterol and hypertriglyceridemia, indicating that sleep disturbances are linked to metabolic abnormalities [35]. This suggests that comprehensive management addressing these factors could improve sleep quality among patients with DM.

There is evidence that T2DM has a greater association with poor sleep quality than T1DM [8,34,36,37]. The results of our study show that adults with T2DM had higher PSQI scores and were poorer sleepers. Birhanu et al. showed that the risk of poor sleep quality increased by 2.16 in patients with T2DM compared to T1DM [34]. Furthermore, Vézina-Im et al. ultimately found a significant association between sleep quality and the type of diabetes, even after adjusting for age and sex [10]. Sleep disorders could contribute to the pathophysiology of T2DM, leading to an increase in the risk for the disease. This could explain the higher rate in these patients [9]. Another explanation is the higher prevalence of complications in patients with T2DM, such as peripheral neuropathy and osteoarthritis, resulting in pain that disturbs sleep. Moreover, the presence of comorbidities such as HTN, dyslipidemia, and obstructive sleep apnea is more common in these patients, which could worsen their sleep quality. Interestingly, we found that hypoglycemia was greater among good sleepers, contradicting the evidence [38]. Nefs et al. showed that hypoglycemia was not associated with poor sleep quality, defined as PSQI > 5 in adults with T1DM and T2DM. However, this was associated with continuous PSQI scores in patients with T2DM [36]. Our finding could be biased, since hypoglycemia was self-reported, which could introduce a margin of error. Furthermore, the availability of DM technology in our practice, such as continuous glucose monitoring systems, could improve sleep quality and reduce the burden of hypoglycemia.

Regarding the impact of sleep quality on glycemic control, Lee et al. performed a systemic review and meta-analysis in patients with T2DM, finding that sleep quality could be an independent risk factor for poor glycemic control. However, they did not firmly conclude this due to publication bias and heterogeneity [12]. Additionally, a similar conclusion was documented in patients with T1DM, which was that good sleep quality reduced HbA1c significantly, more precisely, by 0.16% [39]. We found no association between sleep quality and its components and HbA1c level. Nefs. et al.’s findings, including both types of diabetes, aligned with our results [36]. Additionally, other studies conducted only on patients with T2DM found no association between glycemic control and sleep quality [19,21]. Diabetes management is a complex multifactorial approach, in which many cofounders play a role in metabolic control. The differences in the sociodemographic and clinical characteristics between the studies could explain discrepant results. Furthermore, considering the observational and cross-sectional nature of most of the studies, bias could have been introduced, and these approaches cannot determine causality between glycemic control and sleep quality.

In our study, we did not find any independent associations between the studied variables and sleep quality in a multiple regression analysis. In contrast, in the literature, age, female gender, smoking, drinking alcohol, unemployment, the presence of social stressors, the duration of DM, T2DM, the presence of comorbidities and complications, depression, quality of life, poor glycemic control, obesity, and insulin usage were potential independent factors causing poor sleep quality in patients with DM in various studies [17,21,25,28,29,30,34]. However, causality could not be proven due to the cross-sectional nature of the studies.

This study, while providing valuable insights into the factors influencing sleep quality among adults with DM, has several limitations that warrant consideration. First, the cross-sectional design limits the ability to establish causality between the identified factors and sleep quality. Additionally, the reliance on self-reported data may introduce bias, as participants might under-report or over-report their sleep quality or related behaviors. In this study, we also did not account for all the potential confounders, such as medication types beyond insulin, detailed diet and exercise habits, other social factors, including the number of children for whom each participant acted as a caregiver, and the participants’ history of sleep disorders, such as sleep apnea, periodic limb movement disorder, and restless legs syndrome, which could significantly influence sleep quality. Furthermore, the generalizability of the findings might be restricted due to the specific demographical and geographical characteristics of the study population, and the small size of the study sample, limiting the applicability of the results to broader, more diverse populations.

## 5. Conclusions

In conclusion, this study underscores the high prevalence of poor sleep quality and the intricate relationship between sleep quality and various demographic and clinical factors among adults with DM. The findings highlight that older age, lower educational levels, being married and a housewife, T2DM, and specific comorbidities such as HTN and dyslipidemia are significantly associated with a higher prevalence of poor sleep quality. This suggests that individualized approaches and sleep interventions may be necessary in these groups of patients. The discrepancies between this study and similar research underscore the need for ongoing investigations into the multifaceted nature of sleep disturbances in populations with DM. These insights are crucial for developing tailored interventions to improve sleep quality and, by extension, overall quality of life for patients with DM.

## Figures and Tables

**Table 1:** Patient demographic and clinical characteristics by PSQI score.

Variable	Good Sleeper (*n* = 62)	Poor Sleeper (*n* = 120)	*p*-Value
Age, years	35.58 ± 19.29	43.89 ± 19.95	0.011 ^a^
Biological sex Male, *n* (%) Female, *n* (%)	30 (48.4) 32 (51.6)	61 (50.8) 59 (49.2)	0.754
Marital status Single, *n* (%) Married, *n* (%) Divorced, *n* (%) Widow, *n* (%)	35 (56.5) 24 (38.7) 3 (4.8) 0	41 (34.2) 61 (50.8) 7 (5.8) 11 (9.2)	0.008 ^a^
Education level Illiterate, *n* (%) R&W, *n* (%) Primary, *n* (%) Intermediate, *n* (%) Secondary, *n* (%) Diploma, *n* (%) University, *n* (%)	0 (0) 0 (0) 6 (9.7) 10 (16.1) 20 (32.3) 5 (8.1) 21 (33.9)	14 (11.7) 3 (2.5) 15 (12.5) 9 (7.5) 26 (21.7) 6 (5) 47 (39.2)	0.022 ^a^
Job Working, *n* (%) Student, *n* (%) Housewife, *n* (%) Retired, *n* (%)	19 (30.6) 29 (46.8) 5 (8.1) 9 (14.5)	39 (32.5) 30 (25) 31 (25.8) 20 (16.7)	0.005 ^a^
Job type Full time, *n* (%) Part-time, *n* (%) Shifts, *n* (%) No job, *n* (%)	50 (80.6) 5 (8.1) 0 (0) 7 (11.3)	94 (78.3) 10 (8.7) 2 (1.7) 14 (11.7)	0.784
			
Smoking status Never, *n* (%) Current, *n* (%) Former, *n* (%)	53 (85.5) 5 (8.1) 4 (6.5)	98 (81.7) 9 (7.5) 13 (10.8)	0.628
Cigarettes per day	6.4 ± 4.93	9.79 ± 18.07	0.27
Time spent exercising per week, hours	1.79 ± 0.99	1.77 ± 1.04	0.775
Comorbidities			
HTN, *n* (%)	11 (17.7)	46 (38.3)	0.005 ^a^
Dyslipidemia, *n* (%)	15 (24.2)	51 (42.5)	0.015 ^a^
Pulmonary disease, *n* (%)	2 (3.2)	8 (6.7)	0.334
Thyroid disease, *n* (%)	8 (12.9)	13 (10.8)	0.679
Psychiatric disease, *n* (%)	3 (4.8)	9 (7.5)	0.493
History of DM complications, *n* (%)	19 (30.6)	53 (43.3)	0.096
Insulin usage, *n* (%)	53 (85.5)	95 (79.2)	0.3
Presence of hypoglycemia, *n* (%)	42 (67.7)	63 (52.5)	0.049 ^a^
Type of DM Type 1, *n* (%) Type 2, *n* (%)	41 (66.1) 21 (33.9)	61 (50.8) 59 (49.2)	0.049 ^a^
DM duration, years	12.6 ± 9.54	14.97 ± 9.82	0.11
Cups of coffee per day	2.61 ± 3.68	2.67 ± 2.19	0.08
Last cup of coffee 3 pm, *n* (%) 6–7 pm, *n* (%) 9–10 pm, *n* (%) 12–1 am, *n* (%)	12 (26.1) 15 (32.6) 12 (26.1) 7 (15.2)	24 (21.8) 39 (35.5) 35 (31.8) 12 (10.9)	0.758
HbA1c, %	8.65 ± 1.61	8.66 ± 1.98	0.841

R&W: reading and writing; DM: diabetes mellitus; HTN: hypertension; HbA1c: glycated hemoglobin. ^a^ Statistically significant at 0.05 level of significance.

**Table 2:** PSQI subcomponents distribution.

PSQI Components	
Subjective sleep quality (average score)	1.02 ± 0.77
very good, *n* (%)	44 (23.5)
fairly good, *n* (%)	105 (56.1)
fairly bad, *n* (%)	29 (15.5)
very bad, *n* (%)	9 (4.8)
Sleep latency (average score)	1.49 ± 1.01
<15 min, *n* (%)	32 (17)
16–30 min, *n* (%)	71 (37.8)
31–60 min, *n* (%)	46 (24.5)
> 60 min, *n* (%)	39 (20.7)
Sleep duration (average score)	1.44 ± 1.17
>7 h, *n* (%)	57 (30.2)
6–7 h, *n* (%)	39 (20.6)
5–6 h, *n* (%)	45 (23.8)
< 5 h, *n* (%)	48 (25.4)
Habitual sleep efficiency (average score)	0.62 ± 1.05
>85%, *n* (%)	128 (68.1)
75–84%, *n* (%)	27 (14.4)
65–74%, *n* (%)	9 (4.8)
< 65%, *n* (%)	24 (12.8)
Sleep disturbances (average score)	1.35 ± 0.54
not during the past month, *n* (%)	1 (0.5)
less than once a week, *n* (%)	126 (67)
once or twice per week, *n* (%)	56 (29.8)
three or more times per week, *n* (%)	5 (2.7)
Use of sleep medication (average score)	0.37 ± 0.83
not during the past month, *n* (%)	154 (81.1)
less than once a week, *n* (%)	11 (5.8)
once or twice a week, *n* (%)	19 (10)
three or more times a week, *n* (%)	6 (3.2)
Daytime dysfunction (average score)	1.09 ± 0.9
no problem at all, *n* (%)	57 (29.8)
only a very slight problem, *n* (%)	72 (37.7)
somewhat a problem, *n* (%)	50 (26.2)
a very big problem, *n* (%)	12 (6.3)
Global PSQI score	7.36 ± 3.53

PSQI: Pittsburgh Sleep Quality Index.

**Table 3:** Correlation between glycemic control and PSQI components scores.

PSQI Components	HbA1c	
	Coefficient Correlation	*p* Value
Subjective sleep quality	−0.01	0.91
Sleep latency	−0.12	0.12
Sleep duration	−0.06	0.41
Habitual sleep efficiency	−0.11	0.13
Sleep disturbances	0.07	0.34
Use of sleep medication	−0.1	0.15
Daytime dysfunction	−0.03	0.68
Global PSQI score	−0.1	0.2

PSQI: Pittsburgh Sleep Quality Index; HbA1c: glycated hemoglobin.

**Table 4:** Results of multiple linear regression analysis with PSQI scores.

Variable	Unstandardized B	Std. Error	*p* Value	Standardized Beta	95% Confidence Interval for B	
					Lower	Upper
Age	0	0	0.893	−0.02	−0.01	0.01
Marital status	0.12	0.06	0.053	0.21	0	0.25
Education level	0	0.02	0.829	−0.02	−0.05	0.04
Job	−0.01	0.04	0.789	−0.02	−0.09	0.07
HTN	0.13	0.1	0.192	0.13	−0.07	0.32
Dyslipidemia	0.06	0.09	0.542	0.06	−0.13	0.24
Hypoglycemia	0.1	0.08	0.182	0.11	−0.05	0.25
DM type	−0.06	0.1	0.549	−0.06	−0.25	0.13
HbA1c	0.01	0.02	0.626	0.04	−0.03	0.05

PSQI: Pittsburgh Sleep Quality Index; HTN: hypertension; DM: diabetes mellitus; HbA1c: glycated hemoglobin.

## Data Availability

The data supporting the findings of this study are available from the corresponding author upon reasonable request.

## Supplementary Materials

The following supporting information can be downloaded at: https://www.mdpi.com/article/10.3390/ijerph21111437/s1. Table S1: Patient demographic and clinical characteristics by diabetes mellitus type.
