# Supplementary material for: Sleep Quality Assessment and Its Predictors Among Saudi Adults with Type 1 and Type 2 Diabetes: A Cross-Sectional Study
Source: Int J Environ Res Public Health. 2024 Oct 29;21(11):1437. doi: 10.3390/ijerph21111437 (PMC11594045; doi:10.3390/ijerph21111437)
Supplement: Supplementary file 1 [file ijerph-21-01437-s001.zip › ijerph-3137974-supplementary.pdf]

Supplementary Table S1. Patient demographic and clinical characteristics by diabetes mellitus type

Variable	Overall (*n*=192)	T1DM (*n*=108)	T2DM (*n*=84)	*P* value
Age, years	40.95 ± 20.04	29.82 ± 17.19	55.26 ± 13.22	0.000[a]
Biological sex				
Male, *n* (%)	98 (51)	52 (48.1)	46 (54.8)	0.363
Female, *n* (%)	94 (49)	56 (51.9)	38 (45.2)	
Marital status				
Single, *n* (%)	80 (41.7)	74 (68.5)	6 (7.1)	
Married, *n* (%)	90 (46.9)	26 (24.1)	64 (76.2)	0.000[a]
Divorced, *n* (%)	10 (5.2)	7 (6.5)	3 (3.6)	
Widow, *n* (%)	12 (6.3)	1 (0.9)	11 (13.1)	
Education level				
Illiterate, *n* (%)	15 (7.8)	5 (4.6)	10 (11.9)	
R&W, *n* (%)	3 (1.6)	0 (0)	3 (3.6)	
Primary, *n* (%)	23 (12)	14 (13)	9 (10.7)	0.175
Intermediate, *n* (%)	20 (10.4)	13 (12)	7 (8.3)	
Secondary, *n* (%)	47 (24.5)	27 (25)	20 (23.8)	
Diploma, *n* (%)	13 (6.8)	6 (5.6)	7 (8.3)	
University, *n* (%)	71 (37)	43 (39.8)	28 (33.3)	
Job				
Working, *n* (%)	60 (31.3)	31 (28.7)	29 (34.5)	
Student, *n* (%)	62 (32.3)	59 (54.6)	3 (3.6)	0.000[a]
Housewife, *n* (%)	38 (19.8)	10 (9.3)	28 (33.3)	
Retired, *n* (%)	32 (16.7)	8 (7.4)	24 (28.6)	
Job type				
Full time, *n* (%)	146 (76)	83 (76.9)	63 (75)	
Part-time, *n* (%)	16 (8.3)	11 (10.2)	5 (6)	0.201
Shifts, *n* (%)	2 (1)	2 (1.9)	0 (0)	
No job, *n* (%)	28 (14.6)	12 (11.1)	16 (19)	
Smoking status				
Never, *n* (%)	161 (83.9)	94 (87)	67 (79.8)	0.06
Current, *n* (%)	14 (7.3)	9 (8.3)	5 (6)	
Former, *n* (%)	17 (8.9)	5 (4.6)	12 (14.3)	
Cigarette per day	8.89 ± 15.61	11.78 ± 22.24	6.3 ± 5.64	0.679
Time exercising per week, hours	1.82 ± 1.07	1.84 ± 1.02	1.8 ± 1.14	0.465
Comorbidities				
HTN, *n* (%)	61 (31.8)	15 (13.9)	46 (54.8)	0.000[a]
Dyslipidemia, *n* (%)	70 (36.5)	21 (19.4)	49 (58.3)	0.000[a]
Pulmonary Disease, *n* (%)	11 (5.7)	6 (5.6)	5 (6)	0.907
Thyroid Disease, *n* (%)	23 (12)	16 (14.8)	7 (8.3)	0.17
Psychiatric Disease, *n* (%)	12 (6.3)	6(5.6)	6 (7.1)	0.652
History of DM complications, *n* (%)	76 (39.6)	33 (30.6)	43 (51.2)	0.004[a]
Insulin usage, *n* (%)	156 (81.3)	102 (94.4)	54 (64.3)	0.000[a]
Presence of hypoglycemia, *n* (%)	112 (58.3)	79 (73.1)	33 (39.3)	0.000[a]

DM duration, years	14.06 ± 9.66	12.33 ± 9.24	16.29 ± 9.8	0.005[a]
Cups of coffee per day	2.67 ± 2.81	2.17 ± 1.82	3.31 ± 3.62	0.05
Last cup of coffee				
3 pm, ***n*** (%)	38 (23.3)	18 (20.9)	20 (26)	
6-7 pm, ***n*** (%)	57 (35)	28 (32.6)	29 (37.7)	0.452
9-10 pm, ***n*** (%)	48 (29.4)	30 (34.9)	18 (23.4)	
12-1 am, ***n*** (%)	20 (12.3)	10 (11.6)	10 (13)	
HbA1c, %	8.68 ± 1.91	8.82 ± 1.88	8.51 ± 1.94	0.168
Global PSQI score, ***n*** (%)	7.36 ± 3.53	6.65 ± 3.09	8.28 ± 3.85	0.005[a]

R&W: reading and writing; DM: diabetes mellitus; T1DM: type 1 diabetes mellitus; T2DM: type 2 diabetes mellitus; HTN: hypertension; HbA1c: glycated hemoglobin. [a] Statistically significant at 0.05 level of significance
